# Morphological identification and genetic characterization of *Anopheles stephensi* in Somaliland

**DOI:** 10.1186/s13071-022-05339-y

**Published:** 2022-07-08

**Authors:** Said Ali, Jeanne N. Samake, Joseph Spear, Tamar E. Carter

**Affiliations:** 1 National Malaria Control Program, Ministry of Health Development, Hargeisa, Somaliland; 2 Baylor University, Waco, TX USA

**Keywords:** Malaria, Invasive species, Mitochondrial DNA, Vector-borne disease

## Abstract

**Abstract:**

Malaria control in Somaliland depends on the effective identification of potential malaria vectors, particularly those that may be invasive. The malaria vector *Anopheles stephensi* has been detected in multiple countries in the Horn of Africa (HOA), but data on its geographic distribution and population genetic diversity are incomplete. We implemented a vector surveillance program and performed molecular analysis of *Anopheles* in three urban areas in Somaliland. Our study confirmed the presence of both the invasive *An. stephensi* and the long-established HOA malaria vector *Anopheles arabiensis*. Further analysis of *An. stephensi* genetic diversity revealed three cytochrome oxidase I (*COI*) haplotypes, all of which have been observed in other countries in East Africa and one also observed in South Asia. We also detected the knockdown resistance (*kdr*) L1014F mutation, which is associated with pyrethroid resistance; this finding supports the need for further assessment of the potential for insecticide resistance. The detection of multiple haplotypes previously observed in other regions of East Africa indicates that *An. stephensi* is an established population in Somaliland and likely shares its origin with other newly identified *An. stephensi* populations in East Africa. The detection of genetic diversity in *An. stephensi* in Somaliland provides a basis for future studies on the history of the species in the region and its dispersal throughout East Africa.

**Graphical Abstract:**

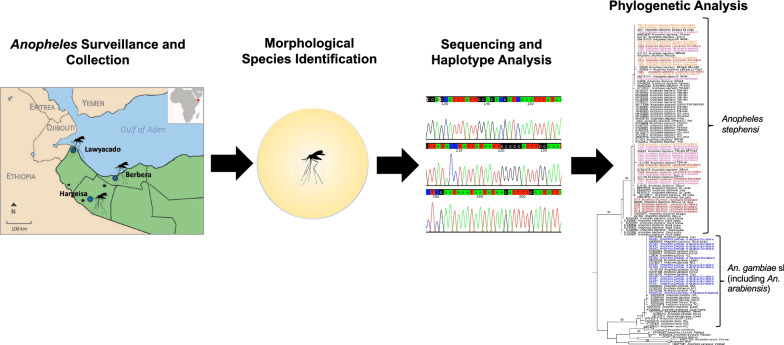

**Supplementary Information:**

The online version contains supplementary material available at 10.1186/s13071-022-05339-y.

## Background

Malaria is a major global health threat, with about 215 million cases reported in Africa in 2019 [1]. While significant progress has been made to reduce malaria cases and mortality over the last decade, the recent detection of a new vector, *Anopheles stephensi,* in the Horn of Africa (HOA) threatens to reverse this progress. *Anopheles stephensi*, a vector found in South Asia and the Middle East, including large parts of the Arabian Peninsula, was first detected in Djibouti in 2012 [2]. Since then, it has been reported in Ethiopia [3], Sudan [4], Somalia [5] and, most recently, Yemen [5].

The presence of *An. stephensi* has raised concern about its spread into urban areas and the potential to increase the number of malaria cases [6–8], given increased urbanization and human movement in Africa. In Asia, *An. stephensi* is one of the few *Anopheles* vectors known to inhabit urban areas [9], likely due to its successful breeding in human-made containers. In addition, studies published between 2019 and 2021 indicate the vector is resistant to pyrethroid-, organophosphate- and carbamate-based insecticides in Ethiopia [10, 11], as observed in established populations (reviewed in Enayati et al. [12]), and is capable of *Plasmodium falciparum* and *Plasmodium vivax* transmission [13, 14]. These findings highlight the growing threat of *An. stephensi* and the need for further investigation of this vector in the Horn of Africa.

Evaluating the risk for malaria transmission by *An. stephensi* requires accurate data on its present distribution in the HOA. In Somaliland, where no earlier records are available on the presence of *An. stephensi*, there was an unusual increase in malaria cases in Sahil, increasing from 87 cases reported in 2019 to 1836 cases reported in 2020 (HMIS/DHIS2). To better understand the basis for this increase, more data on the geographic distribution of the vector populations are needed. *Anopheles gambiae* sensu lato (s.l.) is the major malaria vector in Somaliland, and ecological niche modeling indicates *An. arabiensis* as the likely candidate from the complex [15], although DNA sequences have not been generated to confirm this identification. *Anopheles stephensi* was detected in Bossaso City, Puntland State, northern Somalia, in 2019. In March 2020, *An. stephensi* was detected in Berbera, the main seaport of Somaliland [5], located in the Sahil region. With this initial report of *An. stephensi* in the region, more information on the distribution of the vector in Somaliland is needed to evaluate its potential impact on malaria transmission. In addition, analysis of the genetic diversity of *An. stephensi* can elucidate the relationship between the population in Somaliland, new populations in the HOA and long-established populations outside the HOA. Here we present morphological and molecular confirmation of the presence of *An. stephensi* in Somaliland and a preliminary analysis of its genetic diversity.

## Methods

### Study sites

**Fig. 1:**
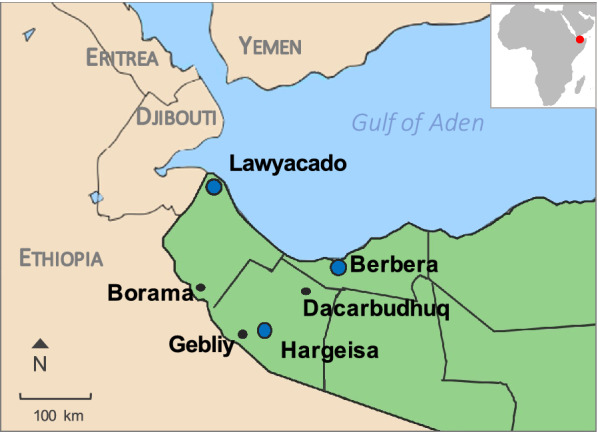
Map of study sites surveyed in Somaliland in 2020. Blue dots represent sites where *Anopheles stephensi* was detected. Map was created using Adobe Illustrator v. 2019 (Adobe Inc., San Jose, CA, USA) and Pages OS 13 (Apple Inc., Cupertino, CA, USA) based on maps from Google Maps (https://www.google.com/maps)

As a follow-up to the initial survey conducted in Berbera in March 2020, we conducted additional surveys between September and November 2020 in six urban sites located in semi-arid or desert climate zones (Fig. 1; Table 1). There are two wet seasons in these areas, from March to July and August to November, respectively, with an average precipitation of 370 mm per year [16]. The elevation in the study sites ranged between 96 and 1500 m a.s.l. We surveyed sites in response to the uptick in the number of reported malaria cases (i.e. in the districts of Hargeisa and Berbera). In the Awdal administrative region (western Somaliland), surveys were conducted in the urban sites of Lawyacado and Borama. Other sites included in this report undergo routine surveillance at fixed sentinel sites on the second or third month of each quarter. In the Marodijeh administrative region, we surveyed the urban areas of Hargeisa (two locations), Dacarbudhuq, and Gebliy. Specimens collected during the survey which initially detected *An. stephensi* in Berbera (Sahil) were also included in the genetic analysis.

**Table 1:** Study sites and collection details from the 2020 survey

Administrative region	District	Study site	Survey type	Altitude (m a.s.l.)	Coordinates
Sahil	Berbera	Wadajir	Single during March 2020	14	N 10.438, E 45.016
Awdal	Lawyacado	Lanta-hawada	Routine surveillance, quarterly	5	N 11.4582, E 43.26313
	Borama	Ali Jawhar	Routine surveillance, quarterly	1468	N 9.94, E 43.18
Marodijeh	Dacarbudhuq	Dacarbudhuq Valley	Routine surveillance, quarterly	948	N 9.86, E 44.53
	Hargeisa	Animal Park	Single from September to October 2020	1266	N 9.5597, E 44.0595
	Hargeisa	Daami	Single from September to October 2020	1268	N 9.5684, E 44.080
	Gebliy	Allaybaday	Routine surveillance, quarterly	1459	N 9.71, E 43.63

### Collections

Specimens were collected during a larval survey using standard dipping techniques. The surveyed breeding habitats included artificial containers or objects, such as discarded tires, metal and plastic tanks, berkads (concrete containers for water storage), and natural water sources, such as freshwater pools and stream margins. We reared larvae in the field insectary using water taken from the breeding sites and baking yeast for feeding and transferred pupae into adult emergence cages. *Anopheles* mosquitoes were identified using the updated key of Afrotropical mosquitoes [17]. The identified *An. stephensi* samples were preserved with silica gel, and a subsample of specimens was sent to Baylor University for molecular analysis.

### PCR and sequencing

DNA was extracted from head and thorax using the Qiagen DNeasy Blood and Tissue kit (Qiagen, Hilden, Germany). Species identification was conducted using the *ITS2* endpoint assay protocol detailed in [18, 19]. The primer sequences for PCR in the *ITS2* endpoint assay are 5.8SB (5′-ATCACTCGGCTCGTGGATCG-3ʹ) and 28SC (5ʹ-GTCTCGCGACTGCAACTG-3ʹ). When the products of the endpoint assay are visualized with gel electrophoresis, a band will be present if the sample contains *An. stephensi* DNA. For further species identification, portions of the mitochondrial cytochrome oxidase subunit I (*COI*) locus and internal transcribed spacer 2 (*ITS2*) locus were PCR amplified and sequenced for subsequent phylogenetic analysis using protocols previously detailed by Carter et al. [20]. The primer sequences for *COI* are LCO1490F (5ʹ-GGTCAACAAATCATAAAGATATTGG-3ʹ) and HCO2198R (5ʹ-TAAACTTCAGGGTGACCAAAAAATCA-3ʹ) [21]. The primer sequences for the *ITS2* sequences are 5.8SB (5ʹ-ATCACTCGGCTCGTGGATCG-3ʹ) and 28SB (5ʹ-ATGCTTAAATTTAGGGGGTAGTC-3ʹ) [19, 22]. The *ITS2* and *COI* sequences were submitted as queries to NCBI BLAST to confirm the correct locus was amplified. Sequences for the haplotypes identified in this study were submitted to NCBI Nucleotide database (Accession nos. ON421572-ON421575).

We also analyzed the pyrethroid resistance-associated voltage-gated sodium channel (*vgsc*) gene for the knockdown resistance mutation (*kdr*). The PCR analyses and sequencing were conducted using previously published protocols [10]. Sequences were submitted as queried to NCBI BLAST to confirm correct *kdr* locus amplification, and *kdr* mutation detection was performed using alignment to reference sequences from Yared et al. [10] in CodonCode Aligner version 8 (CodonCode Corp., Centerville, MA, USA).

### Sequence analysis

For further confirmation of species identification, we performed phylogenetic analysis that incorporated *COI* sequences from the *Anopheles* included in this study, sequences of *An. stephensi* from the HOA, Arabian Peninsula, Middle East and South Asia and representative sequences from the high scoring segment pair sequences retrieved from GenBank via Nucleotide BLAST [23]. Because *COI* is not able to differentiate the different members of *An. gambiae* s.l., additional phylogenetic analysis of the *ITS2* sequence was performed for *An. gambiae* s.l. specimens for species level identification. Phylogenetic relationships were inferred using a maximum likelihood approach with RAxML GUI [24] using the GTR model of nucleotide substitutions and gamma model for rate of heterogeneity (GTRGAMMA option). *Anopheles implexus* was designated as an outgroup for *COI* analysis and *Anopheles christyi* as an outgroup for *ITS2* analysis. The tree with the highest log likelihood was visualized and formatted in FigTree [25].

## Results and discussion

A total of 103 breeding sites across the six sites were inspected during the survey, and larvae were only detected in 28 berkads. Of the six sites surveyed, *An. stephensi* was detected at three sites: Berbera (March 2020), Hargeisa (September 2020) and Lawyacado (October 2020). Mosquito larvae/pupae collected at these three sites were reared to adulthood. No *An. stephensi* were detected at Dacarbudhuq, Borama and Gebliy.

**Fig. 2:**
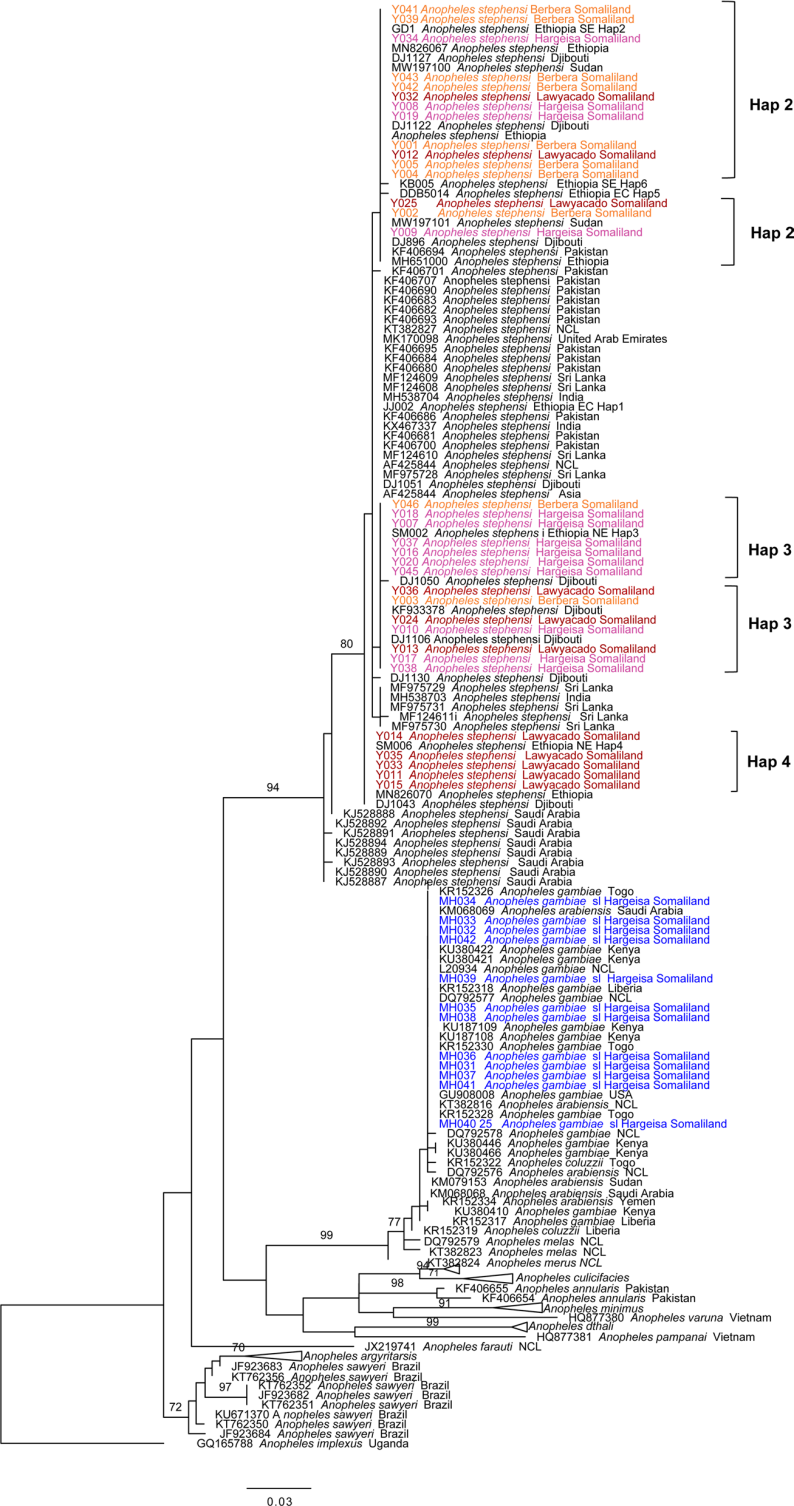
Phylogeny of *COI* sequences based on maximum likelihood approach. Tree with the highest likelihood score (Final ML Optimization Likelihood Optimization: − 1753.729080) is shown. *Anopheles stephensi* sequences are shown in color, indicating location of capture, with red indicating taxa in Lawyacado, orange indicating taxa in Berbera and pink indicating taxa in Hargeisa. For *An. gambiae* sensu lato, blue indicates Hargeisa. Bootstrap values > 70 for notable species clades are shown at nodes. Nodes without numbers had a value < 70. *Anopheles stephensi* COI haplotypes found in Somaliland (Hap 2–4) are identified with brackets. Abbreviations:* COI*, Mitochondrial cytochrome oxidase subunit I locus; Hap, haplotype; ML, maximum likelihood

Forty-eight of the collected specimens were sent to Baylor University for molecular analysis, of which morphological characterization identified 36 as *An. stephensi* and 12 as *An. gambiae* s.l. All *An. gambiae* s.l. specimens were from Hargeisa and were included to confirm that any *An. stephensi* specimen could be correctly distinguished morphologically from the native vector. Molecular analysis of extracted DNA was successful in identifying the species of 45 of the 48 mosquitoes; DNA extraction was unsuccessful in three morphologically identified *An. stephensi*, and thus molecular analysis was not possible for these three mosquitoes. The species identity of the 33 morphologically-identified *An. stephensi* was confirmed based on the presence of the characteristic band in the *ITS2* endpoint assay. All 12 morphologically-identified *An. gambiae* s.l. were confirmed not to be *An. stephensi* based on the absence of the bands in the *ITS2* endpoint assay. Phylogenetic analysis of *COI* further confirmed *An. stephensi* (bootstrap = 94) and *An. gambiae* s.l. (bootstrap = 99) identifications (Fig. 2). Phylogenetic analysis of the *ITS2* sequence in *An. gambiae* s.l. further revealed that the *An. gambiae* s.l. specimens are *An. arabiensis* (bootstrap = 99, Additional file 1: Figure S1). These results are the first genetic confirmation of these species in Somaliland. These findings also confirm successful implementation of the new morphological key and support the continued use of the key for distinguishing *An. stephensi* from *An. gambiae* s.l.

Analysis of the *An. stephensi COI* sequence data indicated three different haplotypes. These haplotypes have been reported in other countries in East Africa, including Ethiopia (Hap 2–3) [14, 26], Djibouti (Hap 2–4) [27] and Sudan (Hap 2) [4]. The multiple shared haplotypes with Ethiopia and Djibouti may indicate a shared origin of *An. stephensi* populations or a pattern of movement of *An. stephensi* between these regions, as would be likely for geographic neighbors. Hap 2 has also been observed outside of the HOA, specifically in Pakistan [28]. The most predominant haplotype was Hap 3 (17/37), followed by Hap 2 (15/37) and Hap 4 (5/37). The distribution of the haplotypes appears to vary by site, although larger sample sizes are needed to confirm this. Notably, *COI*-Hap 4 was only observed in Lawyacado, the most northwestern site and approximately 18 km from Djibouti city. We also noted that Lawyacado has the highest number of observed haplotypes (*n* = 3). This follows the trend observed in Ethiopia, where the highest diversity was observed in the northern sites and those most proximal to the eastern border [29]. Preliminary evidence of differences in the level of diversity and genetic differentiation between sites should be followed up with population genomic analysis.

To obtain preliminary insight into the evolution of the insecticide resistance mutations in *An. stephensi* in this region, we genotyped the *kdr* locus associated with pyrethroid resistance. Of the 32 mosquitoes with available *kdr* sequence data, eight carried the *kdr* L1014F mutation, all as heterozygotes. The highest frequency of *kdr* variants was in Lawyacado (7/10, 70%). Only one of 12 specimens carried the *kdr* L1014F in Hargeisa, and none were observed in Berbera (0/10, 0%). There were no *kdr* L1014S mutations detected in this sample set. The frequency of *kdr* mutations was found to be higher in Lawyacado than what has been reported in other *An. stephensi* populations (e.g. 0–16.7% in eastern Ethiopia) [10, 30], which may reflect differences in local vector control approaches. The high-frequency site, which is close to the Djibouti border, may also relate to heightened insecticide-based vector control in nearby Djibouti. However, the sample size is small in this study, and tests for pyrethroid resistance are needed to determine phenotypic resistance. Additional surveillance is critical to determine the extent of insecticide resistance in *An. stephensi* in Somaliland and to track how *An. stephensi* populations are responding to changes in control approaches.

## Conclusion

Overall, our study confirms the presence of *An. stephensi* in multiple sites in Somaliland. Detected *An. stephensi* showed similar breeding sites as previously reported in other studies in East Africa. Preliminary analysis of *COI* sequence data reveals shared haplotypes within East Africa and abroad, suggesting *An. stephensi* may have spread across country borders. Further genomic analyses will reveal the pattern and direction of spread within the HOA to inform programs aimed at controlling and/or eliminating *An. stephensi* in Somaliland and to prevent further spread in Africa.

## Supplementary Information

**Additional file 1: Figure S1.** Phylogenetic analysis of *ITS2* using the maximum likelihood approach. Tree with the highest likelihood score (Final ML Optimization Likelihood: -981.332474) is shown. *Anopheles gambiae* s.l. from Somaliland are highlighted in blue. Bootstrap values > 70 for notable species clades are shown at nodes. Nodes without numbers had a value < 70.

## Data Availability

All data generated or analyzed during this study are included in this published article and as supplemental files. Sequences are deposited in the NCBI GenBank database (Accession # ON421572-ON421575).
