# Supplementary figures and images for: Morphological identification and genetic characterization of Anopheles stephensi in Somaliland
Source: Parasit Vectors. 2022 Jul 8;15:247. doi: 10.1186/s13071-022-05339-y (PMC9270831; doi:10.1186/s13071-022-05339-y)

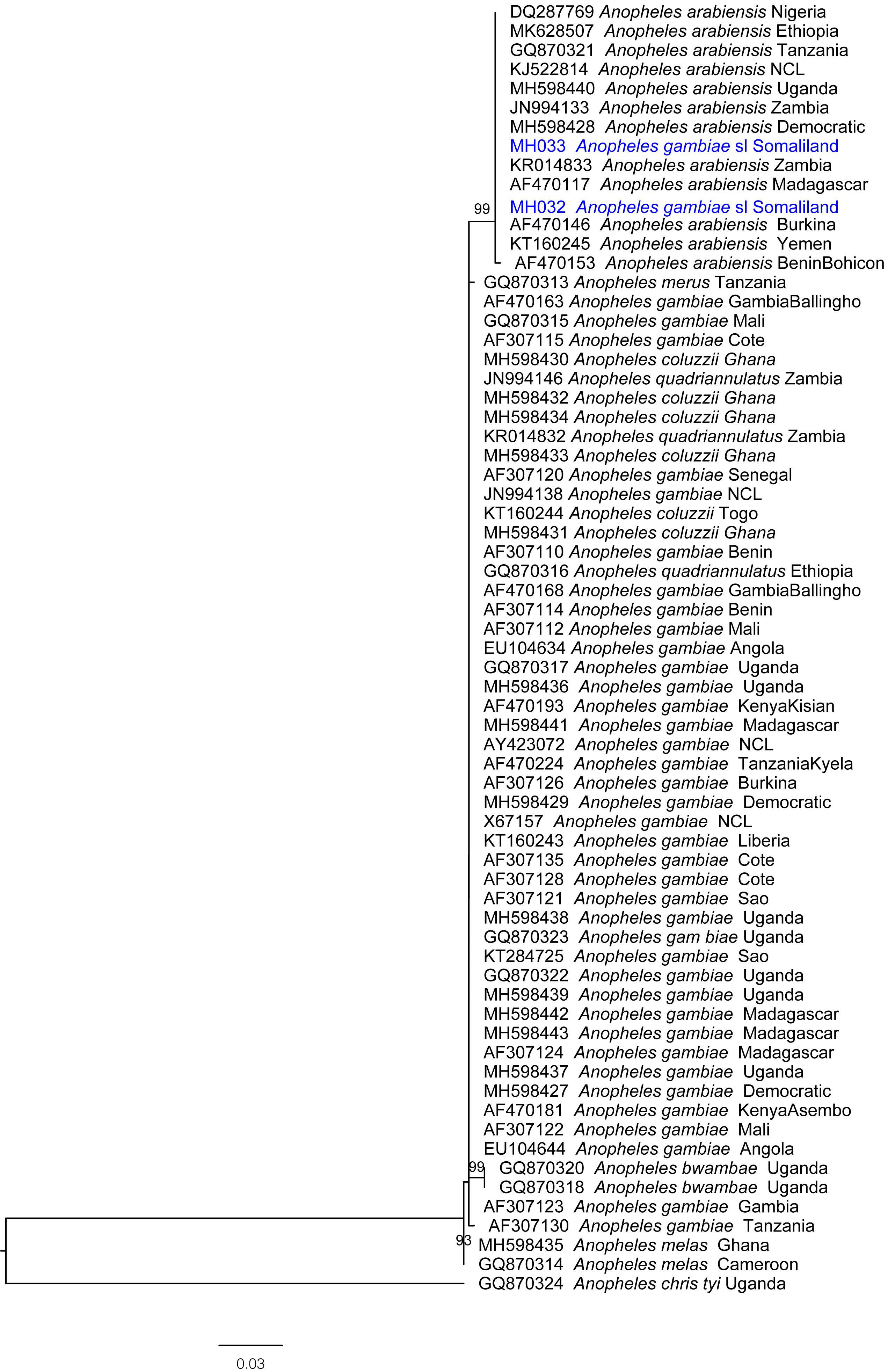

Supplement: Supplementary file 1 — Additional file 1: Figure S1. Phylogenetic analysis of ITS2 using the maximum likelihood approach. Tree with the highest likelihood score (Final ML Optimization Likelihood: -981.332474) is shown. Anopheles gambiae s.l. from Somaliland are highlighted in blue. Bootstrap values > 70 for notable species clades are shown at nodes. Nodes without numbers had a value < 70. [file 13071_2022_5339_MOESM1_ESM.tif]
